# Wilfully out of sight? A literature review on the effectiveness of cancer-related decision aids and implementation strategies

**DOI:** 10.1186/s12911-016-0273-8

**Published:** 2016-03-15

**Authors:** Anne Herrmann, Elise Mansfield, Alix E. Hall, Rob Sanson-Fisher, Nicholas Zdenkowski

**Affiliations:** Priority Research Centre for Health Behaviour, Health Behaviour Research Group, University of Newcastle and Hunter Medical Research Institute, W4, HMRI Building, University Drive, Callaghan, NSW Australia; Department of Medical Oncology, Calvary Mater Newcastle, Waratah, NSW Australia

**Keywords:** Decision aids, Implementation, Neoplasm

## Abstract

**Background:**

There is evidence to suggest that decision aids improve a number of patient outcomes. However, little is known about the progression of research effort in this area over time. This literature review examined the volume of research published in 2000, 2007 and 2014 which tested the effectiveness of decision aids in improving cancer patient outcomes, coded by cancer site and decision type being targeted. These numbers were compared with the volume of research examining the effectiveness of strategies to increase the adoption of decision aids by healthcare providers.

**Methods:**

A literature review of intervention studies was undertaken. Medline, Embase, PsychInfo and Cochrane Database of Systematic Reviews were searched. The search was limited to human studies published in English, French, or German. Abstracts were assessed against eligibility criteria by one reviewer and a random sample of 20 % checked by a second. Eligible intervention studies in the three time periods were categorised by: i) whether they tested the effectiveness of decision aids, coded by cancer site and decision type, and ii) whether they tested strategies to increase healthcare provider adoption of decision aids.

**Results:**

Over the three time points assessed, increasing research effort has been directed towards testing the effectiveness of decision aids in improving patient outcomes (*p* < 0.0001). The number of studies on decision aids for cancer screening or prevention increased statistically significantly (*p* < 0.0001) whereas the number of studies on cancer treatment did not (*p* = 1.00). The majority of studies examined the effectiveness of decision aids for prostate (*n* = 10), breast (*n* = 9) or colon cancer (*n* = 7). Only two studies assessed the effectiveness of implementation strategies to increase healthcare provider adoption of decision aids.

**Conclusions:**

While the number of studies testing the effectiveness of decision aids has increased, the majority of research has focused on screening and prevention decision aids for only a few cancer sites. This neglects a number of cancer populations, as well as other areas of cancer care such as treatment decisions. Also, given the apparent effectiveness of decision aids, more effort needs to be made to implement this evidence into meaningful benefits for patients.

**Electronic supplementary material:**

The online version of this article (doi:10.1186/s12911-016-0273-8) contains supplementary material, which is available to authorized users.

## Background

### Patients as key players in their own healthcare

Over the last two decades cancer care has evolved from a paternalistic, clinician-centred model to a patient-centred model [1, 2]. Patient-centred care places great emphasis on involving patients in their own healthcare [3, 4]. Clinical decision making is now largely viewed as a collaborative process in which the clinician, the patient (and their support persons) choose healthcare options together, based on the patient’s informed preferences [5, 6]. Involving patients in their healthcare decisions is associated with improved patient outcomes, including decreases in patient unmet information needs and anxiety and increases in patients’ satisfaction with the consultation [7, 8]. Shared decision making can improve patients’ quality of life [9–12].

### Preference-sensitive healthcare decisions are challenging

Patients’ willingness to become involved in decisions may be hampered by difficulties in choosing between the various healthcare options available to them [13, 14]. This is especially true for “preference-sensitive” decisions, where there is little or no difference in the medical effectiveness of the available healthcare options. In these instances the final decision involves weighing up the costs and benefits of the different options according to the values and preferences of the patient [3, 15]. With an increasing variety of treatment and care options, more and more cancer prevention, screening and treatment decisions are becoming “preference sensitive.” For example, early-stage breast cancer patients and their clinicians may have a number of different treatment options to choose from, including surgery, cytotoxic or endocrine therapy [16]. Some patients may have the option to decide whether they receive chemotherapy before surgery (neoadjuvant) or after surgery (adjuvant). Each of these treatments shows similar medical effectiveness for these patients but holds various side effects and impacts that may be valued differently by different patients [17].

### Decision aids to help patients make difficult healthcare decisions

To assist patients in making these difficult decisions, healthcare providers have been encouraged to use patient decision aids. Decision aids are interventions which provide patients with specific information on their available options and guide patients towards choosing the option that aligns with their values. They intend to encourage patients to become more involved in the decision making process [18, 19]. Decision aids can be delivered in various formats, such as face-to-face, as written booklets or web-based tools [20]. They cover a variety of healthcare options, including cancer screening, prevention and treatment [21].

### There is evidence for the effectiveness of decision aids

Numerous reviews have provided considerable evidence of the effectiveness of decision aids in improving patient outcomes [22–25]. The first Cochrane review on the effectiveness of decision aids was published in 2001, and concluded that decision aids improve knowledge, reduce decisional conflict, and stimulate patients to be more active in decision making [26]. Updated versions of this review were published in 2003, 2009, 2011 and 2014, which all supported the original findings [20, 27–29]. To date, over 100 Randomized Controlled Trials (RCTs) exist that demonstrate that decision aids are effective in improving patient outcomes. Despite the evidence for the effectiveness of decision aids, they are not commonly used in practice [30]. Previous research has identified barriers and enablers which preclude the implementation of decision aids [31–33]. Little is known about whether the focus of research on the effectiveness of decision aids has changed over time and whether this evidence has translated into the development and testing of strategies to implement decision aids. Once the effectiveness of decision aids in a certain area has been established, research should move from testing the effectiveness of these interventions to testing the effectiveness of implementing decision aids into routine care.

### Research output as measure of research effort

Examining the volume of peer-reviewed research output using bibliometric methods can be used as a proxy indicator of scientific productivity [34–37]. As a result, assessing the volume of research output can provide an indication of the focus of research effort and where future research is needed most. To date, there has been no time sampling of the volume of research examining the effectiveness of decision aids compared to the volume examining the effectiveness of strategies to increase their adoption by healthcare providers. We aimed to give an indication of the focus of research efforts, in order to provide an indication of where future research is required.

## Aims

The aim of this review was to provide a snapshot of where research effort focusing on cancer-related decision aids has been directed to over the last 15 years. We examined changes in the volume of research that examined the effectiveness of cancer-related decision aids, across three time points. We also categorised eligible articles by cancer type and decision being targeted. Finally, we compared the number of studies that assessed the effectiveness of cancer-related decision aids to the number of studies that assessed strategies to increase the adoption of decision aids by healthcare providers.

## Methods

### Literature search

The electronic databases Medline, Embase, PsychInfo and Cochrane Database of Systematic Reviews were searched using the OVID platform. We selected these databases due to their focus on biomedicine and health publications in scholarly journals. The search strategy included three categories of search terms and subject headings: cancer, decision making/decision aids and patient participation. We adapted the search strategy to the requirements of each individual database. The full search strategy for each database is available in Additional file 1. Searches were restricted to English, French and German language publications and human studies. Although most scientific research is published in English, the importance of non-English studies is hard to predict [38, 39]. English, French and German belong to the most common alternative languages used in scientific research [40–42]. Studies published in French or German were included in this review to reduce the likelihood of English language bias. Reference lists of systematic reviews on the effectiveness of decision aids were also searched to ensure that all relevant studies were included in this paper. Where feasible and applicable the PRISMA guidelines were followed [43].

### Inclusion and exclusion criteria

Studies were included if they were intervention studies which examined either: the effectiveness of decision aids on patient outcomes or the effectiveness of strategies to increase provider adoption of patient decision aids. Eligible papers were those published in any country in 2000, 2007 or 2014. These time periods were chosen prospectively as the patient-centred care model gained popularity after the influential report ‘Ensuring Quality Cancer Care’ released by the US National Cancer Board published in 1999, advocating for patient-centred care [2]. Awareness of the patient-centred model was further heightened by the 2001 Institute of Medicine report ‘Crossing the Quality Chasm’ [1]. We excluded case studies, commentaries, conference abstracts, proposed studies, protocol papers and editorials.

### Definitions

We based our definition of patient decision aids on that proposed by the International Patient Decision Aid Standards (IPDAS) Collaboration [44–46]. IPDAS aims to improve the quality and effectiveness of patient decision aids by establishing standards for improving their content, development, implementation, and evaluation [18, 19, 47]. Decision aids were defined as interventions which help patients to participate in making deliberated choices among healthcare options. They explicitly state the decision to be made and provide specific, evidence-based information on the available healthcare options as well as information on the possible risks and benefits of each option. Decision aids aim to help patients to clarify and communicate the value they associate with each option [20, 46]. Strategies to increase healthcare provider adoption of decision aids were defined as any actions taken in order to increase provider usage of decision aids in clinical practice. Implementation strategies were coded as such if they were targeted at the healthcare provider, and/or if they were targeted at the healthcare system.

### Paper coding

After removing the duplicate results, abstracts were screened according to the eligibility criteria by one reviewer (AH). They were rejected if the reviewer determined from the title and abstract that the study did not meet the inclusion criteria. Full text copies of the remaining publications were retrieved and further assessed against the eligibility criteria by the same reviewer (AH). A random sample of 20 % of full text studies identified as eligible were checked for relevance and double-coded by a second reviewer (EM). Eligible studies in the three time periods were categorised by whether they tested: i) the effectiveness of decision aids in improving cancer patients’ outcomes, or ii) the adoption of decision aids by healthcare providers. Studies testing the effectiveness of decision aids were also coded by cancer type of the study sample. The type of decision being targeted was coded as either screening/prevention or treatment. Screening decisions aids include those which assist patients to make a decision about whether they want to undergo cancer screening, such as mammography and colonoscopy. Cancer prevention decision aids include those which assist patients to make a decision about whether they will undergo a procedure to lower the risk of getting cancer, such as prophylactic mastectomy or immunisation. Cancer treatment decision aids include those designed to help patients choose between different cancer treatments.

### Analysis

One way trend tests were performed to examine the changes in the proportions of studies on the effectiveness of decision aids as well as on screening or prevention and treatment decision aids separately across time. Analyses were programmed using Stata v13.0 (StataCorp Ltd, College Station, TX).

## Results

### Search results

**Fig. 1:**
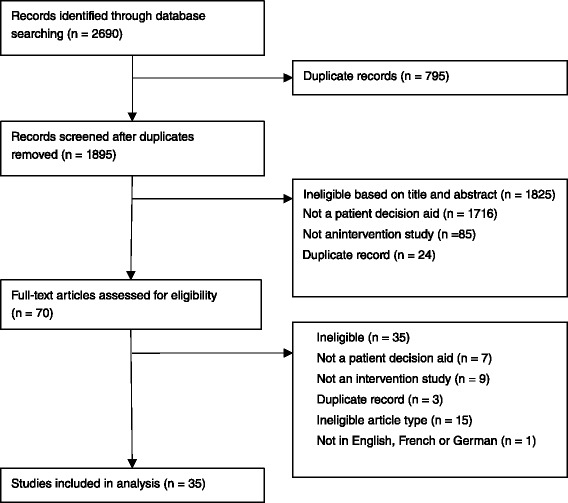
Flow chart of search strategy and study selection, according to the PRISMA guidelines [43]

As shown in Fig. 1, a total of 2,690 citations were retrieved using the search strategy. Of these, 35 full-text studies met the eligibility criteria and were included in this review. Double coding of 20 % of all full-text articles resulted in 100 % agreement between the reviewers (Kappa = 1.000). A list of included citations is provided in Additional file 2.

### Studies reporting on the effectiveness of decision aids

**Fig. 2:**
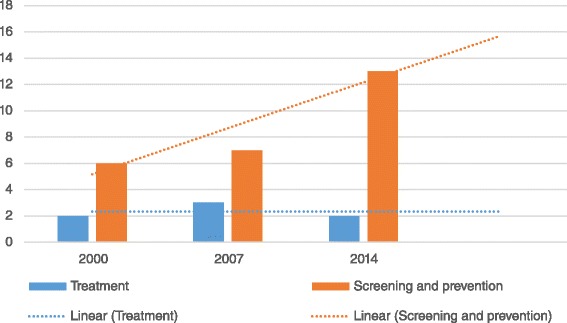
Numbers of studies on the effectiveness of decision aids by decision type being targeted

**Fig. 3:**
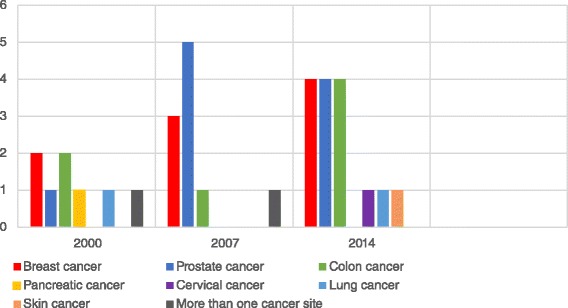
Numbers of studies on the effectiveness of decision aids by cancer site

Of the included studies, 33 tested the effectiveness of decision aids in improving cancer patient outcomes. The number of studies examining the effectiveness of decision aids increased significantly across the three time points (*p* < 0.0001), from 8 studies in 2000 (22.8 %), to 10 studies in 2007 (28.5 %) and 15 studies in 2014 (42.8 %). As shown in Fig. 2, the majority of these papers focused on decision aids for cancer screening and prevention (*n* = 26), compared to those focused on treatment (*n* = 7). Across the three time points assessed, the number of studies focusing on cancer screening and prevention decision aids increased significantly (*p* < 0.0001), while the number focused on cancer treatment did not (*p* = 1.00, Fig. 2). Decision aids were found for breast, prostate, colon, lung, pancreatic, skin, ovarian and cervical cancer. The majority of studies focused on prostate (*n* = 10), breast (*n* = 9) and colon cancer (*n* = 7). Two studies focused on more than one cancer type, including breast, ovarian, cervical and colon cancer (Fig. 3).

### Studies reporting on strategies to implement decision aids

**Fig. 4:**
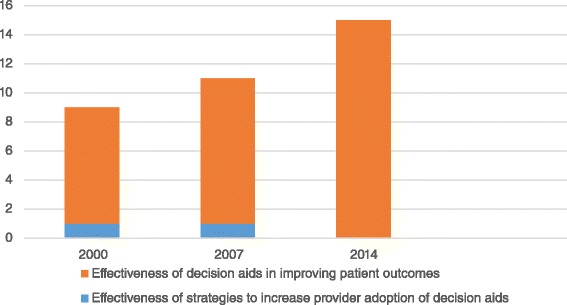
Number of studies on the effectiveness of decision aids compared to the number of studies on implementation strategies

Only the two remaining studies, published in 2000 and 2007, assessed the effectiveness of strategies to increase the implementation of decision aids into clinical practice. Due to the low number of these studies, a statistical comparison was not performed. The number of studies testing the effectiveness of decision aids vs the number of studies examining implementation strategies are reported in Fig. 4.

## Discussion

### Research priorities by relative volume of intervention studies

We examined the progression of research volume which tested the effectiveness of decision aids by cancer site and decision type being targeted, across three time points. Also, we compared these numbers with the volume of research testing the effectiveness of strategies to increase the adoption of decision aids by healthcare providers. Our data suggests that an increase in research effort has been directed towards assessing the effectiveness of decision aids for cancer screening and prevention. The majority of studies focused on prostate, breast and colon cancer. Only two studies examined the effectiveness of strategies to increase provider adoption of decision aids, despite evidence illustrating the benefit of decision aids’ for some patient outcomes [20, 25].

### Lack of research on the effectiveness of decision aids for cancer treatment

Although decision aids are available for a number of healthcare decisions, research has been increasingly focusing on screening and prevention decisions as opposed to treatment decisions. One reason for the larger volume of screening and prevention decision aids may be that these interventions are aimed at healthy people, rather than a vulnerable patient group. This can facilitate the research process, for example by easier access to large sample sizes and by the facilitation of the ethical review process. Developing and testing decision aids on treatment options needs considerable clinical input, which relies on strong collaborations between researchers and clinicians [48, 49]. For example, clinicians may vary in their preferences for different treatment options based on their clinical experience [50]. If clinicians disagree in the content of a decision aid, the development of such decision aids might be hindered [32, 51]. However, treatment decisions can be very distressing for patients [13]. Also, as the number of treatment options available to patients has been increasing, particularly in relation to “preference sensitive” treatments, opportunities arise to develop and test decision aids for cancer treatment decisions.

### Narrow research focus on decision aids for only a few cancer types

Over the last 15 years, increasing research effort has been directed towards examining the effectiveness of decision aids on prostate, breast and colon cancer. This may seem understandable as according to the latest GLOBOCAN statistics these are amongst the most prevalent cancer types worldwide [52]. Screening recommendations for breast, colon and prostate cancer have been established for decades which could further explain the increased research volume focused on these sites [53]. However, research with other cancer types where decision aids could be beneficial seems to be sparse. For instance, there are guideline recommendations for cervical cancer screening, prevention and treatment, which could motivate decision aid research in this area [54, 55]. But a lack of such research across these three time periods has been shown. Also, lung cancer has high incidence and burden, but little research exists about decision aids for lung cancer screening, prevention and treatment [20, 25]. This might be because there are no nationally standardised screening programmes for lung cancer in many countries as there are for other types of cancer, such as breast or colon [53, 56, 57]. However, many lung cancer patients are faced with difficult healthcare decisions, such as a choice between different treatment modalities. Some of these require the patient to decide between a slightly higher chance of longer survival or fewer treatment related side-effects [58, 59]. Thus there is a need for effective decision aids for cancer populations other than prostate, breast or colon.

### Lack of research effort towards testing effective implementation strategies

This review has shown that the research volume on decision aids for cancer screening and prevention has increased over the three time points assessed. Given that decision aids are not commonly used in practice [30], it may be expected that we should have started to see the testing of strategies to implement decision aids that have been shown to be effective. However, we found only two studies on the effectiveness of implementation strategies across the three time periods assessed. The little attempt to translate evidence into meaningful benefits for patients may result from various factors, such as methodological difficulties of carrying out well-controlled implementation trials; perception that optimal care is already being delivered; difficulties of addressing further barriers to the adoption of decision aids in practice; and potential further questions to be answered by ongoing research on the effectiveness of decision aids. These factors are discussed below.

#### Methodological difficulties of carrying out implementation trials

Implementation of decision aids may involve changes in processes of care. This necessitates system-orientated change, which is not always amenable to the “gold-standard” RCT intervention design. Decision aids are complex interventions in a complex field of social interactions. They address various influences on behaviour. Attention should be paid to this complexity and to the context of implementation [24, 60]. It has been argued that RCTs are not suitable for taking into account all relevant contextual factors in which complex interventions are delivered and received [61]. The randomization and blinding required by RCTs cannot always accommodate the complexity and flexibility needed to test these interventions on a system level [62, 63]. According to the Medical Research Council's guidance for evaluating complex interventions, a range of alternate study designs should be considered, including Stepped Wedge or Multiple Baseline Designs [64, 65]. Future attempts to test implementation strategies should consider these designs. As planning and conducting such complex trials takes an extended period of time it may be that much of the implementation research is still being carried out [66]. It is possible that we see a surge in such studies in the near future.

#### Perception that optimal care is already being delivered

There may be an assumption that evidence-based strategies are already being used in practice. For example, O’Brien and colleagues reported that some clinicians have high confidence in their own communication skills and believe that patients understand the information they have conveyed [31]. Clinicians in this study have indicated that decision aids’ effects on the decision making process are not compelling enough to change their practice. Consequently, some have argued that there is no need to conduct research to implement decision aids into routine care [31]. However, given the increasing range and availability of prevention, screening and treatment options, healthcare decisions have become increasingly difficult. Especially in clinical situations where there is low or conflicting evidence on the medical effectiveness of the available healthcare options it is crucial to involve patients’ preferences in the decision making process.

#### Further barriers to the adoption of decision aids in practice

Findings of previous research indicate that clinicians identify numerous barriers that affect their ability to implement patient decision aids [31–33, 67]. Such barriers include: concerns about how comprehensive and current the content of decision aids is, lack of awareness of existing decision aids, time constraints, and concerns about how to integrate decision aids into clinicians’ workflow [32, 68]. Designing implementation strategies to overcome these barriers is challenging. There is little evidence that passive dissemination through strategies such as guidelines is effective [69]. Implementation strategies need to actively target healthcare providers, patients or both [66]. They should be tailored to the specific setting avoiding “one-fits-all-solutions”. Instead of controlling for confounding variables, implementation attempts need to investigate these variables in order to better understand the long-term implementation of decision aids [70]. Practice-based research within the real world setting of daily cancer care needs to be conducted [71]. Researchers should focus on illuminating processes, rather than “package” and use the strengths of collaborative research across various contexts in order to systematically study the impact of the individual settings [70].

#### Open questions regarding the effectiveness of decision aids

Although there is a large body of evidence demonstrating that decision aids are effective in improving a range of patient outcomes, open questions remain in regards to the stated effectiveness. For example, further studies are required which explore the “active ingredients” of decision aids and clinically relevant outcomes apart from the ones already assessed [24]. Greater understanding of the mechanisms of action of decision aids and further evidence for their clinical impact may increase their acceptability in clinical practice and motivate more attempts to design and evaluate implementation strategies. Further open questions remain in regards to the “orientation” and “insight” phase of implementing decision aids into practice. Consequently, we need further in-depth investigation of clinicians’ understanding and opinion on decision aids before we ask them to implement these tools [23, 51, 72, 73]. However, as the body of work on the effectiveness of decision aids has been growing, we hope that the number of intervention studies which test implementation strategies will develop accordingly.

### Limitations

The results of this study should be considered in light of several limitations. First, only three years of publication were included in this study. It is possible that the trends in research output differ in the years which were not assessed. In addition, due to the low numbers of eligible studies, it was not possible to compare statistically the trends in effectiveness and implementation trials over time. This limits the strength of our conclusions about the relative increase in effectiveness compared with implementation trials. However, the inclusion of these three time points provides an indication of research effort over the past 15 years. Grey literature such as policy documents and dissertations were not included as they do not meet the standards associated with peer-reviewed publications. It is possible that the exclusion of such research has biased the results due to the file drawer problem, whereby studies showing null (or negative) findings tend not to be published. The exclusion of conference abstracts may have led to underestimating the number of implementation studies currently underway.

## Conclusions

Although multiple Cochrane reviews provide evidence that decision aids are effective in improving a range of patient outcomes, our review suggests that research testing the effectiveness of decision aids has increased over the three time points assessed. Research effort in this area has focused predominantly on screening and prevention decisions in only a few cancer sites. This neglects a number of cancer populations, as well as other areas of cancer care such as treatment decisions. Further, once the effectiveness of certain decision aids is established, strategies to increase their adoption by healthcare providers need to be designed and tested. Such research will help to ensure that the benefits of decision aids reach the intended patient populations.

## Additional files

Additional file 1: Search strategies for each database. (DOCX 13 kb) Additional file 2: List of citations for included studies. (DOCX 16 kb)
