# Supplementary material for: Wilfully out of sight? A literature review on the effectiveness of cancer-related decision aids and implementation strategies
Source: BMC Med Inform Decis Mak. 2016 Mar 15;16:36. doi: 10.1186/s12911-016-0273-8 (PMC4793751; doi:10.1186/s12911-016-0273-8)
Supplement: Additional file 1: — Search strategies for each database. (DOCX 13 kb) [file 12911_2016_273_MOESM1_ESM.docx]

### Additional file 1 – Search strategies for each database

Database: MEDLINE 1946 to Present with Daily Update

Search Strategy:

--------------------------------------------------------------------------------

1 exp neoplasms/ (2746146)

2 decision support techniques/ (13910)

3 decision aid*.tw. (1427)

4 2 or 3 (14668)

5 decision making, computer assisted/ (2561)

6 decision making/ or choice behavior/ (93958)

7 5 or 6 (96449)

8 physician patient relations/ (62989)

9 patient education as topic/ (73272)

10 patient participation/ or patient preference/ (22845)

11 8 or 9 or 10 (148799)

12 7 and 11 (9869)

13 4 or 12 (23918)

14 1 and 13 (3840)

15 limit 14 to (humans and (editorial or letter or news)) (307)

16 14 not 15 (3533)

17 limit 16 to yr="2001" (88)

18 limit 16 to yr="2007" (136)

19 limit 16 to yr="2014" (354)

20 17 or 18 or 19 (578)

Database: Embase Classic+Embase <1947 to 2015 August 05>

Search Strategy:

--------------------------------------------------------------------------------

1 exp neoplasm/ (3787744)

2 decision support system/ (15551)

3 decision aid*.tw. (2142)

4 exp decision making/ (237828)

5 patient/ or cancer patient/ (1717880)

6 patient education/ (91855)

7 patient participation/ (18909)

8 patient satisfaction/ (95412)

9 doctor patient relation/ (84354)

10 5 or 6 or 7 or 8 or 9 (1970972)

11 4 and 10 (36134)

12 2 or 3 (17172)

13 11 or 12 (52281)

14 1 and 13 (9691)

15 limit 14 to (human and (book or book series or editorial or letter or note)) (747)

16 14 not 15 (8944)

17 limit 16 to yr="2001" (118)

18 limit 16 to yr="2007" (232)

19 limit 16 to yr="2014" (1244)

20 17 or 18 or 19 (1594)

Database: PsycINFO <1806 to July Week 3 2015>

Search Strategy:

--------------------------------------------------------------------------------

1 exp neoplasms/ or cancer*.tw. (51282)

2 decision support systems/ (2450)

3 decision aid*.tw. (966)

4 2 or 3 (3298)

5 exp decision making/ (72923)

6 "shared decision making".tw. (1440)

7 client participation/ (1489)

8 5 or 6 (73401)

9 7 and 8 (277)

10 4 or 9 (3552)

11 1 and 10 (296)

12 limit 11 to human (289)

13 limit 12 to yr="2001" (1)

14 limit 12 to yr="2007" (17)

15 limit 12 to yr="2014" (23)

16 13 or 14 or 15 (41)

Search Name: Decision making

Date Run: 10/08/15 04:14:42.540

Description:

ID Search Hits

#1 MeSH descriptor: [Neoplasms] explode all trees 54477

#2 MeSH descriptor: [Decision Support Techniques] explode all trees 3251

#3 decision aid* 2974

#4 #2 or #3 5935

#5 MeSH descriptor: [Decision Making, Computer-Assisted] explode all trees 3740

#6 MeSH descriptor: [Decision Making] explode all trees 2767

#7 MeSH descriptor: [Choice Behavior] explode all trees 904

#8 #5 or #6 or #7 6479

#9 MeSH descriptor: [Physician-Patient Relations] explode all trees 1105

#10 MeSH descriptor: [Patient Education as Topic] explode all trees 6735

#11 MeSH descriptor: [Patient Education as Topic] explode all trees 6735

#12 MeSH descriptor: [Patient Participation] explode all trees 902

#13 MeSH descriptor: [Patient Preference] explode all trees 372

#14 #9 or #10 or #11 or #12 or #13 8473

#15 #8 and #14 512

#16 #4 or #15 6305

#17 #1 and #16 810
