# Supplementary material for: Wilfully out of sight? A literature review on the effectiveness of cancer-related decision aids and implementation strategies
Source: BMC Med Inform Decis Mak. 2016 Mar 15;16:36. doi: 10.1186/s12911-016-0273-8 (PMC4793751; doi:10.1186/s12911-016-0273-8)
Supplement: Additional file 2: — List of citations for included studies. (DOCX 16 kb) [file 12911_2016_273_MOESM2_ESM.docx]

### Additional file 2 – List of included studies

1. Brundage MD, Feldman-Stewart D, Dixon P, Gregg R, Youssef Y, Davies D et al. A treatment trade-off based decision aid for patients with locally advanced non-small cell lung cancer. Health Expect. 2000;3(1):55-68. doi:10.1046/j.1369-6513.2000.00083.x.

2. Clark S, Bluman LG, Borstelmann N, Regan K, Winer EP, Rimer BK et al. Patient motivation, satisfaction, and coping in genetic counseling and testing for BRCA1 and BRCA2. J Genet Couns. 2000;9(3):219-35. doi:10.1023/A:1009463905057.

3. Domenighetti G, Grilli R, Maggi JR. Does provision of an evidence-based information change public willingness to accept screening tests? Health Expect. 2000;3(2):145-50. doi:10.1046/j.1369-6513.2000.00081.x.

4. Holmes-Rovner M, Valade D, Orlowski C, Draus C, Nabozny-Valerio B, Keiser S. Implementing shared decision-making in routine practice: Barriers and opportunities. Health Expect. 2000;3(3):182-91. doi:10.1046/j.1369-6513.2000.00093.x.

5. Lawrence VA, Streiner D, Hazuda HP, Naylor R, Levine M, Gafni A. A cross-cultural consumer-based decision aid for screening mammography. Prev Med. 2000;30(3):200-8. doi:10.1006/pmed.1999.0620.

6. Pignone M, Harris R, Kinsinger L. Videotape-based decision aid for colon cancer screening. A randomized, controlled trial. Ann Intern Med. 2000;133(10):761-9+I16.

7. Schapira MM, Vanruiswyk J. The effect of an illustrated pamphlet decision-aid on the use of prostate cancer screening tests. J Fam Pract. 2000;49(5):418-24.

8. Sepucha KR, Belkora JK, Tripathy D, Esserman LJ. Building bridges between physicians and patients: results of a pilot study examining new tools for collaborative decision making in breast cancer. J Clin Oncol. 2000;18(6):1230-8.

9. Wolf AM, Schorling JB. Does informed consent alter elderly patients' preferences for colorectal cancer screening? Results of a randomized trial. J Gen Intern Med. 2000;15(1):24-30.

10. Davison BJ, Goldenberg SL, Wiens KP, Gleave ME. Comparing a generic and individualized information decision support intervention for men newly diagnosed with localized prostate cancer. Cancer Nurs. 2007;30(5):E7-15.

11. Feldman-Stewart D, Brennenstuhl S, Brundage MD. A purpose-based evaluation of information for patients: an approach to measuring effectiveness. Patient Educ Couns. 2007;65(3):311-9.

12. Krist AH, Woolf SH, Johnson RE, Kerns JW. Patient education on prostate cancer screening and involvement in decision making. Ann Fam Med. 2007;5(2):112-9.

13. Lin JW, Chu PL, Liou JM, Hwang JJ. Applying a multiple screening program aided by a guideline-driven computerized decision support system - A pilot experience in Yun-Lin, Taiwan. J Formos Med Assoc. 2007;106(1):58-68.

14. Mathieu E, Barratt A, Davey HM, McGeechan K, Howard K, Houssami N. Informed choice in mammography screening: a randomized trial of a decision aid for 70-year-old women. Arch Intern Med. 2007;167(19):2039-46.

15. Metcalfe KA, Poll A, O'Connor A, Gershman S, Armel S, Finch A et al. Development and testing of a decision aid for breast cancer prevention for women with a BRCA1 or BRCA2 mutation. Clin Genet. 2007;72(3):208-17.

16. Ozanne EM, Annis C, Adduci K, Showstack J, Esserman L. Pilot trial of a computerized decision aid for breast cancer prevention. Breast J. 2007;13(2):147-54.

17. Ruffin MTt, Fetters MD, Jimbo M. Preference-based electronic decision aid to promote colorectal cancer screening: results of a randomized controlled trial. Prev Med. 2007;45(4):267-73.

18. Stalmeier PF, van Tol-Geerdink JJ, van Lin EN, Schimmel E, Huizenga H, van Daal WA et al. Doctors' and patients' preferences for participation and treatment in curative prostate cancer radiotherapy. J Clin Oncol. 2007;25(21):3096-100.

19. Wakefield CE, Meiser B, Homewood J, Peate M, Kirk J, Warner B et al. Development and pilot testing of two decision aids for individuals considering genetic testing for cancer risk. J Genet Couns. 2007;16(3):325-39. doi:10.1007/s10897-006-9068-x.

20. Weinrich SP, Seger R, Curtsinger T, Pumphrey G, NeSmith EG, Weinrich MC. Impact of pretest on posttest knowledge scores with a Solomon Four research design. Cancer Nurs. 2007;30(5):E16-28.

21. Alden DL. Decision aid influences on factors associated with patient empowerment prior to cancer treatment decision making. Med Decis Making. 2014;34(7):884-98. doi:10.1177/0272989X14536780.

22. Carney PA, Lee-Lin F, Mongoue-Tchokote S, Mori M, Leung H, Lau C et al. Improving colorectal cancer screening in Asian Americans: Results of a randomized intervention study. Cancer. 2014;120(11):1702-12. doi:10.1002/cncr.28640.

23. Clouston K, Katz A, Martens PJ, Sisler J, Turner D, Lobchuk M et al. Does access to a colorectal cancer screening website and/or a nurse-managed telephone help line provided to patients by their family physician increase fecal occult blood test uptake?: Results from a pragmatic cluster randomized controlled trial. BMC Cancer. 2014;14:263. doi:10.1186/1471-2407-14-263.

24. Davis SN, Sutton SK, Vadaparampil ST, Meade CD, Rivers BM, Patel MV et al. Informed decision making among first-degree relatives of prostate cancer survivors: A pilot randomized trial. Contemp Clin Trials. 2014;39(2):327-34. doi:10.1016/j.cct.2014.10.007.

25. Hersch J, Jansen J, Barratt A, Irwig L, Houssami N, Jacklyn G et al. Overdetection in breast cancer screening: development and preliminary evaluation of a decision aid. BMJ Open. 2014;4(9):e006016. doi:10.1136/bmjopen-2014-006016.

26. Lafata JE, Cooper G, Divine G, Oja-Tebbe N, Flocke SA. Patient-physician colorectal cancer screening discussion content and patients’ use of colorectal cancer screening. Patient Educ Couns. 2014;94(1):76-82. doi:10.1016/j.pec.2013.09.008.

27. Miller SM, Roussi P, Scarpato J, Wen KY, Zhu F, Roy G. Randomized trial of print messaging: the role of the partner and monitoring style in promoting provider discussions about prostate cancer screening among African American men. Psychooncology. 2014;23(4):404-11. doi:10.1002/pon.3437.

28. Schonberg MA, Hamel MB, Davis RB, Griggs MC, Wee CC, Fagerlin A et al. Development and evaluation of a decision aid on mammography screening for women 75 years and older. JAMA Intern Med. 2014;174(3):417-24. doi:10.1001/jamainternmed.2013.13639.

29. Shaffer VA, Tomek S, Hulsey L. The effect of narrative information in a publicly available patient decision aid for early-stage breast cancer. Health Commun. 2014;29(1):64-73. doi:10.1080/10410236.2012.717341.

30. Sie AS, van Zelst-Stams WA, Spruijt L, Mensenkamp AR, Ligtenberg MJ, Brunner HG et al. More breast cancer patients prefer BRCA-mutation testing without prior face-to-face genetic counseling. Fam Cancer. 2014;13(2):143-51. doi:10.1007/s10689-013-9686-z.

31. Thomas R, Glasziou P, Rychetnik L, Mackenzie G, Gardiner R, Doust J. Deliberative democracy and cancer screening consent: A randomised control trial of the effect of a community jury on men’s knowledge about and intentions to participate in PSA screening. BMJ Open. 2014;4(12):e005691. doi:10.1136/bmjopen-2014-005691.

32. Volk RJ, Linder SK, Leal VB, Rabius V, Cinciripini PM, Kamath GR et al. Feasibility of a patient decision aid about lung cancer screening with low-dose computed tomography. Prev Med. 2014;62:60-3. doi:10.1016/j.ypmed.2014.02.006.

33. Watts KJ, Meiser B, Wakefield CE, Barratt AL, Howard K, Cheah BC et al. Online prostate cancer screening decision aid for at-risk men: A randomized trial. Health Psychol. 2014;33(9):986-97. doi:10.1037/a0034405.

34. Wegwarth O, Kurzenhauser-Carstens S, Gigerenzer G. Overcoming the knowledge-behavior gap: The effect of evidence-based HPV vaccination leaflets on understanding, intention, and actual vaccination decision. Vaccine. 2014;32(12):1388-93. doi:10.1016/j.vaccine.2013.12.038.

35. Wilkins T, Gillies RA, Panchal P, Patel M, Warren P, Schade RR. Colorectal cancer risk information presented by a nonphysician assistant does not increase screening rates. Can Fam Physician. 2014;60(8):731-8.
